# Global patterns in marine organic matter stoichiometry driven by phytoplankton ecophysiology

**DOI:** 10.1038/s41561-022-01066-2

**Published:** 2022-11-21

**Authors:** Keisuke Inomura, Curtis Deutsch, Oliver Jahn, Stephanie Dutkiewicz, Michael J. Follows

**Affiliations:** 1 Graduate School of Oceanography, University of Rhode Island, Narragansett, RI USA; 2 School of Oceanography, University of Washington, Seattle, WA USA; 3 Department of Earth, Atmospheric and Planetary Sciences, Massachusetts Institute of Technology, Cambridge, MA USA; 4 Department of Geosciences and High Meadows Environmental Institute, Princeton University, Princeton, NJ USA

**Keywords:** Element cycles, Microbial ecology, Microbial biooceanography

## Abstract

The proportion of major elements in marine organic matter links cellular processes to global nutrient, oxygen and carbon cycles. Differences in the C:N:P ratios of organic matter have been observed between ocean biomes, but these patterns have yet to be quantified from the underlying small-scale physiological and ecological processes. Here we use an ecosystem model that includes adaptive resource allocation within and between ecologically distinct plankton size classes to attribute the causes of global patterns in the C:N:P ratios. We find that patterns of N:C variation are largely driven by common physiological adjustment strategies across all phytoplankton, while patterns of N:P are driven by ecological selection for taxonomic groups with different phosphorus storage capacities. Although N:C varies widely due to cellular adjustment to light and nutrients, its latitudinal gradient is modest because of depth-dependent trade-offs between nutrient and light availability. Strong latitudinal variation in N:P reflects an ecological balance favouring small plankton with lower P storage capacity in the subtropics, and larger eukaryotes with a higher cellular P storage capacity in nutrient-rich high latitudes. A weaker N:P difference between southern and northern hemispheres, and between the Atlantic and Pacific oceans, reflects differences in phosphate available for cellular storage. Despite simulating only two phytoplankton size classes, the emergent global variability of elemental ratios resembles that of all measured species, suggesting that the range of growth conditions and ecological selection sustain the observed diversity of stoichiometry among phytoplankton.

## Abstract (web summary)

Ecosystem modelling suggests that a range of growth conditions and ecological selection of phytoplankton explain global patterns of C:N:P ratios in marine organic matter.

## Main

Nearly a century ago, Alfred C. Redfield described the relationship between the average elemental ratios of phytoplankton and biogeochemical cycles^1^. Since then, the ‘Redfield ratios’ (C:N:P = 106:16:1) have become a cornerstone of oceanography. They affect carbon export and storage in the deep ocean and thus atmospheric CO_2_ concentration^2–4^, and the intensity of the oxygen minimum zones^5^. The elemental ratios of nutrient demand by phytoplankton^6^ affect microbial resource competition, regulating the balance between denitrification and nitrogen fixation and the global N inventory^7–9^. While the plasticity of elemental ratios in phytoplankton populations has long been recognized, fixed Redfield ratios continue to be widely used in ecological and biogeochemical models^10–12^.

Regional variation of elemental ratios in organic matter production and export has been observed in the ratios of surface nutrient drawdown^13^ and detected in large-scale geochemical tracer distributions^5,14,15^. The latitudinal gradients inferred from geochemical data have been confirmed by global compilations of direct measurements of bulk organic matter, supporting a strong latitudinal variation of N:P and P:C in organic matter, and weaker trends in N:C^16^. Together, these observations reveal substantial correlations between elemental stoichiometry and phytoplankton community structure, with high N:P and P:C in subtropical regions dominated by small phytoplankton^14^, and lower ratios elsewhere. While the stoichiometries of bulk organic matter and residual surface nutrients probably originate from phytoplankton, the potential underlying physiological and ecological mechanisms have not been elucidated.

Laboratory studies have revealed substantial variations in elemental proportions of phytoplankton within and across taxa^17–20^, both of which could underlie large-scale patterns of stoichiometric variation. Intra-taxonomic variations reflect the balance of major macromolecular pools, having distinct elemental ratios^19,21,22^. While carbon occurs in most macromolecules, nitrogen is predominantly associated with proteins, and phosphorus is largely contained in RNA and storage molecules^19,21^. The allocation to protein and RNA increases with growth rate^20–23^, enabling faster biosynthesis and leading to higher N and P relative to C. Thus, phytoplankton in a fast-growing environment (for example, high nutrient) are expected to have high N:C and P:C^24,25^. Intracellular storage can also have a large impact on elemental ratios, especially for P, for which the structural pools are much smaller than those of N and C^21^. Differences in resource allocation at the cellular scale represent a physiological mechanism by which global stoichiometric patterns can arise from depth and/or spatial variations in the growth conditions of phytoplankton.

There is also a distinct pattern in inter-taxonomic variation of the elemental ratios. Measurements across multiple taxa show that N:P ratio of eukaryotes is on average lower than that of prokaryotes^26^. The difference can be partly explained by the capacity to hold excess phosphorus^24^. Ocean regions with a higher fraction of larger eukaryotic phytoplankton may lead to lower N:P than regions dominated by smaller prokaryotes^27^, provided enough P is available. Large-scale differences in the size structure of phytoplankton communities introduce additional ecological mechanisms that may generate global stoichiometric patterns^26^, modulating the physiological factors that arise from cellular-scale allocation.

The biological causes of observed large-scale distributions of organic matter C:N:P remain poorly understood. In particular, the relative contribution of plasticity within phytoplankton groups versus ecological selection among groups with systematic differences in stoichiometric ratios is not known. Theoretical studies of variable stoichiometry typically employ the internal-stores model^27,28^, which is empirically informed but does not resolve the macromolecular allocation or its acclimation to changing environmental conditions. Models that relate macromolecular allocation to the physiological status of the organism provide more mechanistic detail (for example, refs. ^25,29–32^, with reviews by refs. ^33,34^ and the supplement of ref. ^21^) but their implications for global stoichiometric patterns have yet to be fully analysed. Here we address this gap by implementing a cellular resource allocation model within a global model of ocean circulation and biogeochemistry. Model simulations reproduce observed variability at the relevant scales, from cells to biomes, allowing an empirically validated diagnosis of key physiological and ecological factors.

## Simulating cellular macromolecules in a global ocean model

**Extended Data Fig. 1:**
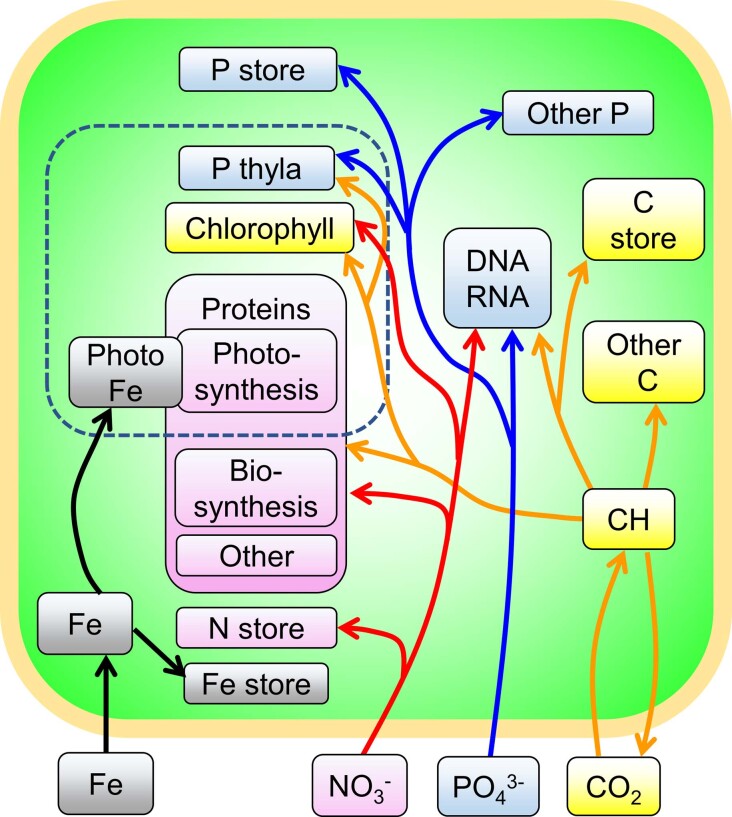
Schematic of CFM-Phyto component of this study. Orange, red, blue, and black arrows represent C, N, P, Fe fluxes, respectively. Yellow, red, blue, gray squares are molecules that mainly influence C, N, P and Fe budgets, respectively. Dashed squre represents photosynthetic molecules. CH and P thyla represent precursor carbohydrates and P in thylakoid membranes, respectively. Green space and orange outline represent the intracellular space and cell membrane layers, respectively.

**Extended Data Fig. 2:**
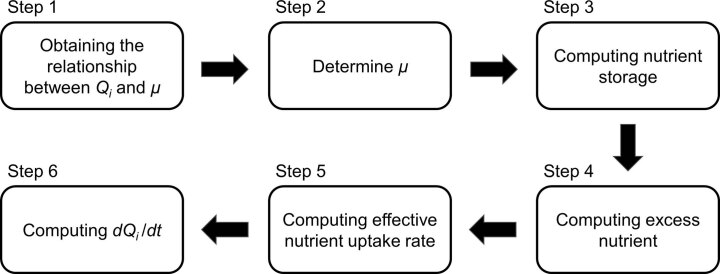
Solution flow of CFM-Phyto part of the modeling. In CFM-Phyto, cellular elemental quotas *Q*_*i*_ and growth rate *μ* are linked by macromolecular allocation.

We incorporated an explicit representation of macromolecular allocation by phytoplankton (CFM-Phyto^21^) into a global general circulation and biogeochemical model, MITgcm^35–37^ (here the combined model is termed MITgcm-CFM; see Methods, Extended Data Figs. 1 and 2, and Extended Data Table 1). The cellular model relates macromolecular and elemental allocation to light intensity and growth rate, and has been calibrated and validated with laboratory data from several species of phytoplankton^21^. The ocean model simulates nutrient and carbon cycles driven by multiple processes, including the growth and mortality of phytoplankton, the formation and decay of particulate and dissolved organic matter^10,38^, and transport by the ocean’s general circulation^35–37^.

The model’s biological component, CFM-Phyto, predicts the elemental stoichiometry of phytoplankton based on resource allocation under different light intensities and nutrient concentrations. Essential elements are apportioned to major groups of macromolecules (for example, proteins, carbohydrates, lipids, DNAs and RNAs) with distinct stoichiometric ratios. The model predicts relationships between the abundance of these molecules, growth rate and light intensities (see Methods). It reproduces laboratory-measured relationships between these factors and cellular stoichiometry that are shared across multiple species of both eukaryotes and prokaryotes^23,39,40^.

The implementation of CFM-Phyto in the MITgcm is adapted from its original form in two ways. First, inter-specific variation is represented by two size classes of phytoplankton: ‘small’ to represent prokaryotes and ‘large’ to represent eukaryotes. Informed by empirical data, large (eukaryotic) phytoplankton are assumed to have a higher P storage capacity^26^ and higher maximum growth rates^41,42^ than small (prokaryotic) phytoplankton (see ‘Differentiating small and large phytoplankton’ in Methods). Second, the elemental allocation is expanded to include two intracellular pools of Fe: Fe in photosystems and Fe in storage (see ‘Relating Fe quota and growth rate’ in Methods).

## Cellular-scale variations

**Fig. 1:**
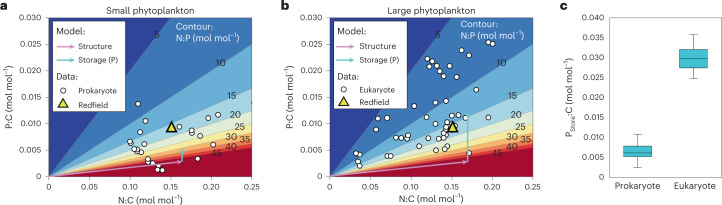
Observed and modelled elemental ratios in phytoplankton. **a**,**b**, Variations in elemental ratios in ‘small’ (prokaryotic) phytoplankton (**a**) and ‘large’ (eukaryotic) phytoplankton (**b**). Colour shading indicates N:P, computed as the ratio of N:C (*x* axis) and P:C (*y* axis). Laboratory data for small prokaryotic cells (white points, **a**) and eukaryotic cells (white points, **b**) at a variety of growth rates and light intensities (excluding a few outliers) (see Supplementary Data and references there). Arrows indicate the stoichiometric ratios predicted by the allocation model decomposed into structural and storage components based on average nutrient and light conditions from the surface ocean at 50° S, where N and P nutrients are largely replete. Lilac arrows indicate the modelled contribution from acclimation in the absence of P storage. Observed points fall above those lines due to P storage, the sense of which is indicated by the light blue vectors (modelled P storage). Larger, eukaryotic cells in **b** are associated with higher storage contributions (longer blue vector) than smaller prokaryotic cells in **a**. **c**, Differences in P storage between small and large plankton size classes in the model are based on empirical estimates derived from laboratory studies of prokaryotic^20,39^ and eukaryotic^44^ phytoplankton (*n* = 43 and 28 for prokaryotes and eukaryotes, respectively. A few data with excess growth-limiting nutrients at the steady state^39^ are not included). The box represents median (centre line) and first and third quartiles (box). The whiskers represent the value range without outliers (those outside the box by 1.5 times the interquartile range). The P storage is estimated based on the differences in P:C under N and P limitations for closest growth rates.

**Extended Data Fig. 3:**
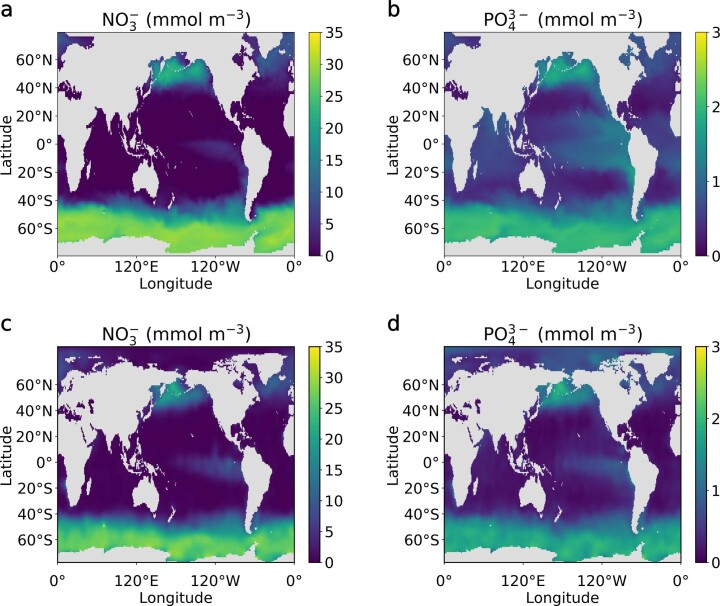
Annual mean NO_3_^−^ and PO_4_^3−^ concentrations at the surface. (a)(b) are model output. (c)(d) climatological average from World Ocean Atlas^69^.

**Extended Data Fig. 4:**
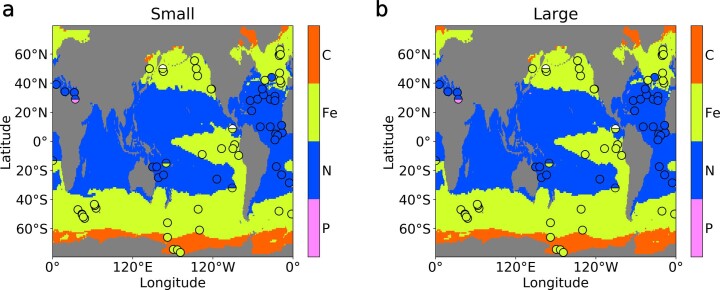
Model-emergent and observed nutrient limitations for phytoplankton growth. (a) Small phytoplankton. (b) Large phytoplankton. Color field shows the limiting nutrient diagnosed from MITgcm. Circles are observed primary nutrient limitation from field data compilation^43^, which did not include an assessment for C limitation.

Global patterns of nutrient uptake yield distributions of surface macronutrient concentrations and of the growth-limiting nutrient that are consistent with observations (Extended Data Figs. 3 and 4)^43^. The model predicts substantial variation in element ratios between small and large plankton, which are consistent with the observed differences between eukaryotes and prokaryotes (Fig. 1). These differences are most pronounced in ratios involving P^27^. Differences in P storage explain most of the P:C and N:P variability in CFM-Phyto (turquoise arrows in Fig. 1a,b). To a smaller degree, the differences also reflect the structural composition (molecules other than P storage) of the cell, which is dominated by proteins. However, N storage has a relatively minor effect on the size-based differences, as implied by the similarity of N:C distribution between prokaryotes and eukaryotes (Fig. 1a,b). Variations in N:C are instead driven by allocation to protein in response to environmental factors.

To quantify the role of P storage in generating large differences in N:P among plankton size classes, we estimated the level of the excess phosphorus uptake for both prokaryotic^20,39^ and eukaryotic^44^ phytoplankton in laboratory experiments. Under N-limited conditions, cells accumulate storage P due to excess P availability and increasing P:C. Under P limitation, cells maintain a minimum, necessary P content in, for example, nucleic acids with a lower P:C. This difference manifests as stored P (per C) associated with luxury uptake^24^. Laboratory studies reveal substantially higher P storage capacity in eukaryotic cells (Fig. 1c), contributing to the lower overall N:P observed among large cells (compare Fig. 1a and b).

## Spatial patterns of elemental ratios

**Fig. 2:**
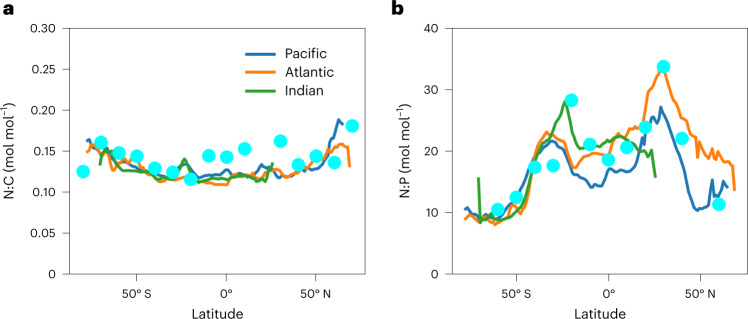
Latitudinal variation of N:C and N:P ratios of bulk organic matter in each basin. **a**, N:C ratio. **b**, N:P ratio. Cyan points are averaged data^52,53^ (data distribution in Extended Data Fig. 5). Curves are model predictions: blue, Pacific Ocean; orange, Atlantic Ocean; green, Indian Ocean. In each panel, a data point with the highest uncertainty is not included. The CV based on the data and model are 0.11 and 0.11 for N:C and 0.33 and 0.29 for N:P, respectively, implying stronger latitudinal variations for N:P.

The observed latitudinal variation in the N:C of organic matter is distinct from that of N:P^16^ (Fig. 2). The N:C ratio has relatively small variation (the coefficient of variation (CV) = 0.11) but increases slightly towards higher latitudes. In contrast, N:P varies strongly (CV = 0.33) and systematically with latitude across all ocean basins; low in the high latitudes, high in the subtropical gyres and intermediate values near the Equator. The values are slightly higher in the Northern Hemisphere, especially in the Atlantic Ocean. These broad-scale, meridional patterns are reproduced by the simulations (Fig. 2). These model–data comparisons are based on particulate organic matter (POM), including phytoplankton biomass, which accounts for a substantial fraction of the modelled standing stock of POM. Thus, the model fidelity to data suggests that the elemental ratios of total organic matter are largely controlled by phytoplankton with only limited alteration from organic matter recycling through the microbial food web.

**Extended Data Fig. 6:**
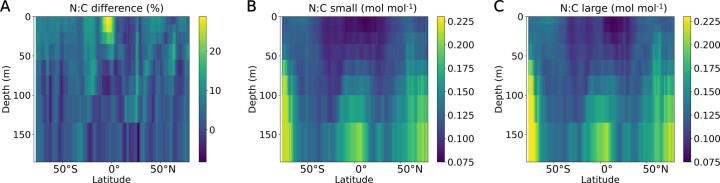
Differences in N:C between small phytoplankton and large phytoplankton. (A) The difference in percent. (B) N:C in small phytoplankton. (C) N:C in large phytoplankton.

The N:C of modelled organic matter pools is strongly influenced by the physiological acclimation of phytoplankton, which are the ultimate source and substantial component of the standing stock of the total particulate detritus. Thus, we seek to interpret the patterns of POM by considering the controls on phytoplankton stoichiometry. Differences in N:C between the size classes are small (Fig. 1 and Extended Data Fig. 6), whereas variations within each size class are large and driven by acclimation (Extended Data Fig. 6). The N:C ratio varies with the primary environmental factors that govern growth rate: nutrient availability and light. Low light requires a high investment in photosynthetic proteins to support growth. In the surface polar oceans, relief of nitrogen stress enables higher growth rates and allocation to biosynthetic and photosystem proteins^21^, leading to high N:C of primary producers. In surface subtropical waters, nitrogen availability limits growth rates, reducing investment in biosynthetic protein, while high light intensity reduces investment in light-harvesting proteins, leading to low protein allocation and low N:C of primary producers. However, deeper in the subtropical water column, lower light and higher nutrient concentrations favour greater investment in protein and modelled N:C increases with depth.

**Fig. 3:**
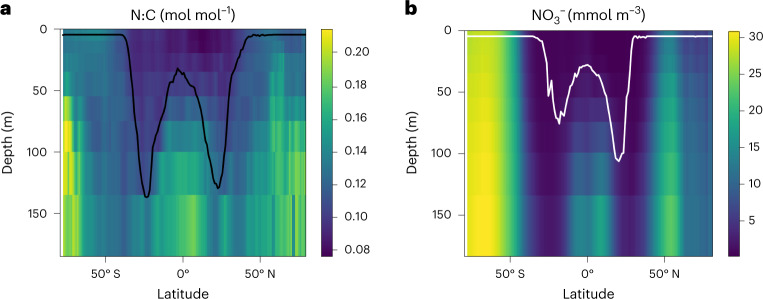
Depth–latitude variation of predicted N:C and NO_3_^−^. **a**,**b**, Longitudinally averaged N:C of phytoplankton (**a**) and NO_3_^−^ concentrations (**b**). The black curve in **a** indicates the depth of phytoplankton biomass maximum. The white curve in **b** indicates the depth of the highest growth rate of the small phytoplankton, which dominate the oligotrophic regime. The values are zonally averaged. Though few in number, such observed growth rate profiles do reveal subsurface maxima^45,54–56^.

Despite the strong variation of modelled N:C with latitude and depth (Fig. 3), the depth-averaged trends in N:C across latitude are relatively small (Fig. 2) because of vertical trends in N:C and biomass. A substantial fraction of the modelled, depth-integrated biomass is associated with a subsurface maximum at low latitudes (Fig. 3), as has been observed in some subtropical profiles^45–48^. The subsurface chlorophyll and biomass maxima arise in part from the trade-off between opposing vertical gradients of nutrients and light^49^. At the depth of the emergent phytoplankton biomass maximum, phytoplankton N:C thus exhibits little variation with latitude (Fig. 3), except at some subpolar latitudes where Fe is limiting (Extended Data Fig. 4), and the modelled latitudinal variation in the depth-averaged N:C (CV = 0.11) is weaker than at the surface (CV = 0.17).

**Fig. 4:**
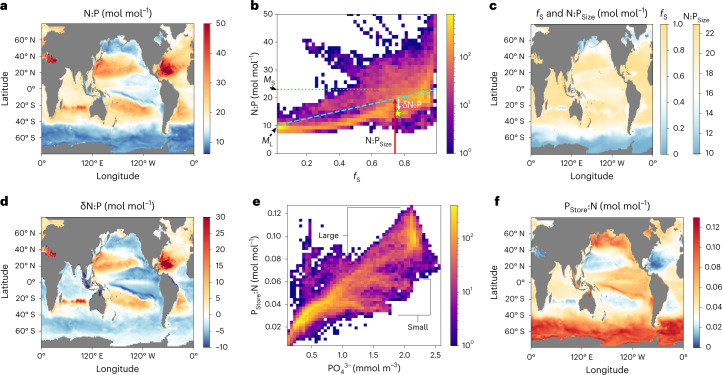
Global distribution of modelled N:P in phytoplankton and its ecological and physiological origins. **a**, N:P of total phytoplankton biomass. **b**, The relationship between total N:P and the fraction of phytoplankton biomass in the small size class (*f*_S_). **c**, The global mean N:P for small and large phytoplankton (*M*_S_ and *M*_L_, labelled on *y* axis in **b**) are used to estimate the N:P variation, N:P_Size_, due to ecological selection for plankton size classes, which has a global pattern that is linearly related to *f*_S_ (see equation (1)). **d**, The residual N:P variation (δN:P) represents the physiological acclimation within size classes, including variations in P storage. **e**, Relationship between P_Store_:N for different PO_4_^3−^ concentrations. **f**, Spatial pattern of P storage per cellular N. All mapped values are ratios of biomass N and P integrated between 0 and 260 m depth.

The latitudinal variation in N:P predicts higher values in the low latitudes and lower N:P in high latitudes with stronger variation (CV = 0.29) than for N:C (CV = 0.11), consistent with particulate observations (Fig. 2) and inferences from nutrient distributions^14^. The model also predicts substantial N:P differences between ocean basins (Fig. 4a). In contrast to N:C, there are substantial differences in N:P between size classes, caused primarily by the distinct P storing capacity between small and large phytoplankton (Fig. 1). In the model, phytoplankton traits are guided by allometric constraints so selective pressures in the oligotrophic regimes favour the smaller size class with lower P storage capacity (Fig. 1). At the same time, even though P is in excess over much of the subtropical ocean, the low concentrations cause accumulation in P storage to be lower than the full potential. Thus, the patterns of total biomass N:P are caused by both physiological acclimation of each size class to its local light and nutrient levels, and by the ecological selection of the dominant size class in each environment.

The model also predicts a high fraction of total P in cellular storage (Fig. 1a,b), which thus plays an important role in setting the overall stoichiometry. In turn, storage capacity is linked to cell size. How much of the N:P variation can be explained by the distribution of plankton size classes? The answer to this question can be estimated from the local fraction of total biomass and respective stoichiometries associated with each size class (Fig. 4c), according to:

1 $${{{\mathrm{N}}}}:{{{\mathrm{P}}}}_{{{{\mathrm{Size}}}}} = f_{\mathrm{S}}M_{\mathrm{S}} + \left( {1 - f_{\mathrm{S}}} \right)M_{\mathrm{L}}$$

where *M*_S_ and *M*_L_ are global mean N:P ratios within each size class (Fig. 4b) and *f*_S_ is the fraction of phytoplankton biomass in the small size class. Variations in N:P_Size_ reflect the global scale pattern of N:P (Fig. 4a,c) and account for about half of the total difference between subtropical and subpolar regimes.

Intra-taxonomic variation of N:P due to acclimation also contributes substantially to the subtropical enhancement and drives a north–south asymmetry. The variation that cannot be explained by plankton size classes is here quantified by δN:P = N:P − N:P_Size_ (Fig. 4b,d). This component of N:P variability is inversely related to the distribution of PO_4_^3−^ (Extended Data Fig. 3), because high PO_4_^3−^ concentrations lead to enhanced P storage (equations (32)–(34) and (25) in the Methods). Surface PO_4_^3−^ is more depleted in the northern than the southern subtropical gyres (Extended Data Fig. 3b) and limits cellular storage of phosphorus regardless of size class (Fig. 4e,f). This results in higher N:P in the Northern Hemisphere subtropics (particularly North Atlantic) than in the southern (Figs. 2b and 4a).

Cellular P storage also explains the asymmetry between polar oceans of the northern and southern hemispheres. Even though large phytoplankton are dominant in high latitudes in both hemispheres (Fig. 4c), surface PO_4_^3−^ concentrations are higher in the Southern Ocean than in the North Atlantic (Extended Data Fig. 3b). This leads to a higher accumulation of plankton P storage (Fig. 4e,f) and lower particulate N:P in the southern high latitudes (Figs. 2b and 4a). The effect of P storage also explains the observed correlation between organic P:C and PO_4_^3−^ concentration^4^. In contrast, model simulations do not predict a hemispheric N:C asymmetry, nor is it evident in observations^16^. Thus, we hypothesize that hemispheric asymmetry in N:P is created mostly by the level of P storage per cellular C rather than variations of N per cellular C. Further investigation is needed to clarify the form of P storage; a large part of it may be polyphosphate, which can account for a substantial fraction of cellular P in diatoms^19^, although RNA or P-containing lipids may also contribute.

## Global diversity of elemental ratios

**Fig. 5:**
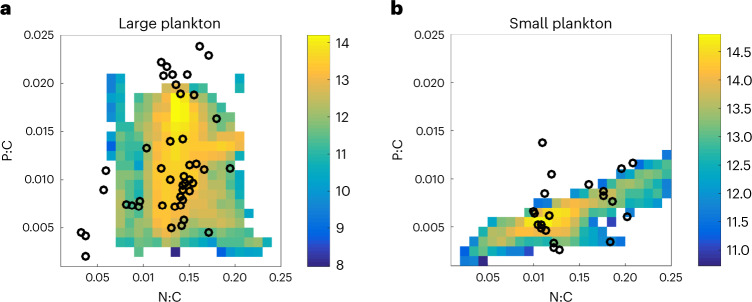
Stoichiometric variability among phytoplankton in modelled populations and species observations. **a**,**b**, Model output of total model phytoplankton biomass (shaded, log_10_ scale in mmol C) is binned according to its local N:C and P:C ratios (mol mol^−1^) alongside the laboratory observations of elemental ratios (points). Large (**a**) and small (**b**) phytoplankton compared against data for eukaryotic and prokaryotic phytoplankton, respectively. The structure of the stoichiometric variability is an emergent property of the model, but broadly consistent with the observed range of traits and the differences between large and small size classes.

**Extended Data Fig. 7:**
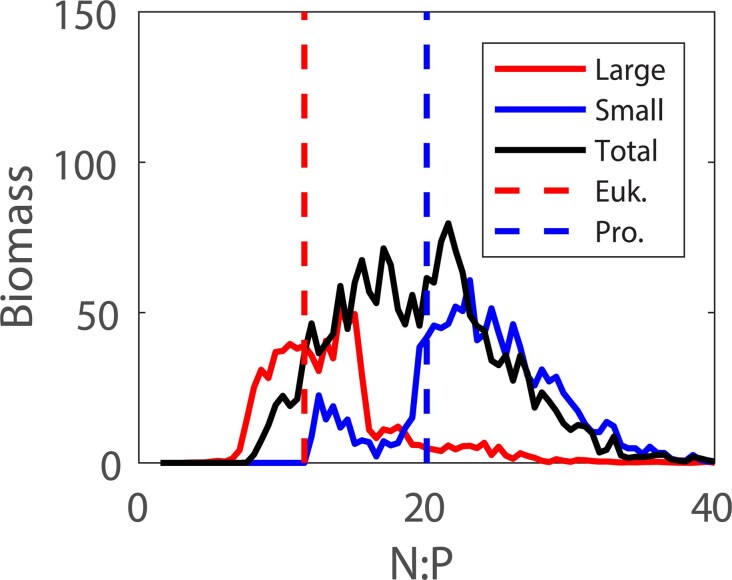
N:P variability among phytoplankton in modeled population and species observations. Model output of total model phytoplankton biomass (Gmol C) is binned according to its local N:P (mol mol^−1^). Distinct curves of biomass versus elemental ratios are separately plotted for small (blue) and large (red) size classes, and compared against the median values of traits observed for small (prokaryote, blue) and large (eukaryote, red).

On a global scale, modelled phytoplankton span a wide range of stoichiometric ratios (Fig. 5), despite the explicit representation of only two plankton size classes. While the preponderance of emergent plankton biomass occurs with a stoichiometry near the canonical Redfield proportions (N:P = 16), each of the elemental ratios exhibits an approximately fourfold range across modelled populations (Fig. 5). This variability across populations closely resembles the observed patterns of stoichiometric ratios sampled across plankton species in laboratory data (Fig. 1). Moreover, the stoichiometric differences between small and large plankton classes are similar to the differences in the median observed species traits between small photosynthetic prokaryotes and larger eukaryotes. The peak modelled biomass of small plankton occurs at a P:C ratio of ~0.05, less than half that of large plankton. A similar difference is also observed between the median values of prokaryotic versus eukaryotic plankton species (Fig. 5). In contrast, the ranges of highest biomass for N:C are similar between small and large as in the data (Fig. 5) because N storage is small relative to the structural N pool in the cell^21^. Similar to P:C ratios, and again consistent with observations, the N:P ratios of model phytoplankton populations have divergent median values between large and small plankton (Extended Data Fig. 7).

Although the model only coarsely resolves phytoplankton size classes, and does not explicitly represent variation at a species level, it nevertheless predicts a wide range of stoichiometric ratios within and between distinct biomes. This stoichiometric diversity and its associated spatial patterns emerge from the physiological acclimation to different environmental conditions, and the ecological selection for populations that are well adapted to those conditions. The similarity between the observed and emergent elemental ratios suggests that the combination of physiological and ecological selection represented in this simple model may be important selective pressures that generate and sustain the diversity of stoichiometric ratios observed among global phytoplankton species.

There are finer-scale taxonomic variations of elemental stoichiometry^18^ than represented here, for example, the high N:P of diazotrophs, particulate inorganic carbon associated with coccolithophores and the intimate connection of diatoms (which contribute to the large, high-storage class) with the silica cycle. Given the broad qualitative success of the simple model presented here, we hypothesize that additional taxonomic resolution and the impact of top-down controls have next-order impacts on C:N:P. This remains to be tested in future studies.

## Implications for plankton stoichiometric diversity

Our results provide a bridge between the recognized diversity and plasticity of plankton stoichiometry at cellular scales, and the coherent large-scale patterns of stoichiometry documented in nutrient distributions and bulk marine organic matter. The consistency between phytoplankton C:N:P predicted by a physiologically based ecosystem model and particulate observations supports the hypothesis that the resource allocation by phytoplankton is the primary influence on the composition of the material that is ultimately removed from the photic zone, and exerts long-term controls on the coupling of biogeochemical cycles. Evaluating the potential for heterotrophic processes to decouple the stoichiometry of exported organic matter from that of phytoplankton^50,51^ will ultimately require simultaneous stoichiometric data on the various living and detrital pools.

Our results suggest that phytoplankton N:C and N:P are controlled by distinct physiological and ecological factors, indicating that elemental ratios cannot simply be modelled with the conventional approach that considers all the elements as uniform pools in a similar way across species^33^. In particular, the modest latitudinal gradient of N:C, despite its high variability at cellular and population scales, reflects physiological acclimation to local environmental conditions through resource allocation. In contrast, the large latitude gradients in P:C and N:P highlight the key role of phosphorus storage and PO_4_^3−^ availability^27^, and the ecological selection for or against plankton communities whose size structure can accommodate large P storage.

While our model considers only two explicit size classes of plankton, it nevertheless captures a wide range of stoichiometric ratios that approximates the range observed among species in laboratory measurements. This stoichiometric diversity arises naturally from the range of growth conditions that occurs in the ocean model. The similarity of the simulated and observed ranges of stoichiometries and the measured differences among distinct taxa suggests that similar selective processes may be responsible for generating and sustaining stoichiometric diversity exhibited by phytoplankton species in the modern ocean.

The model presented here can be used to predict and interpret macromolecular distributions in phytoplankton biomass in the ocean and provides a physiological framework for predicting biological and ecological responses to climate change. Given that the elemental ratios of phytoplankton influence the export of nutrients and carbon to the deep ocean, mechanistic representations of plankton resource allocation may be essential in understanding long-term responses to and feedbacks between marine elemental cycles, the carbon cycle and climate.

## Methods

We incorporated a macromolecular model of phytoplankton (CFM-Phyto) into the global ocean model (MITgcm). This combined model predicts cellular growth rate based on the macromolecular allocation, which in turn is used to determine the elemental stoichiometry of phytoplankton for the next model time step.

The phytoplankton component of the model is executed using the following algorithm, which is illustrated graphically in Extended Data Fig. 2: (1) relate the growth rate and elemental stoichiometry of phytoplankton based on the macromolecular allocation; (2) evaluate the possible growth rates under four different limiting nutrient assumptions and select the lowest rate: Liebig’s Law of the Minimum; (3) evaluate storage of non-limiting elements; (4) evaluate excess of non-limiting elements relative to maximum quotas; (5) based on that excess, evaluate effective nutrient uptake rate; and (6) evaluate the change in the elemental stoichiometry based on the balance between the growth rate and effective nutrient uptake rate. We describe the procedural details in the following text. Parameter values are listed in Extended Data Table 1. See ref. ^21^ for further details and justification of each equation in CFM-Phyto; here we repeat equations essential to explain the model used in the current study.

### Connecting the elemental stoichiometry and the growth rate

The first step of the algorithm is to obtain the relationship between the current elemental stoichiometry and the growth rate (*μ*). To do that, we use CFM-Phyto^21^ (Extended Data Fig. 1). The model is based on the assumption of pseudo-steady state with respect to macromolecular allocation; in other words, the cellular-scale acclimation occurs rapidly relative to environmental changes. Laboratory studies show that macromolecular re-allocation occurs on the timescale of hours to days^19^. This is fast relative to the rates of environmental change in our coarse-resolution ocean simulations and so steady state solutions^21^ are used to relate growth rate, macromolecular allocation and elemental stoichiometry, as described in detail below. We first describe the case of N quota (here defined as *Q*_N_; moles cellular N per mole cellular C) in detail, and then we briefly explain the case of P and C quotas as the overall procedures are similar. After that, we describe the case with Fe quota, which extends the previously published model^21^ for this study.

### Relating N quota and growth rate

CFM-Phyto describes the allocation of N quota as follows, focusing on the quantitatively major molecules:

2 $$Q_{\mathrm{N}} = Q_{\mathrm{N}}^{{\mathrm{Pro}}} + Q_{\mathrm{N}}^{{\mathrm{RNA}}} + Q_{\mathrm{N}}^{{\mathrm{DNA}}} + Q_{\mathrm{N}}^{{\mathrm{Chl}}} + Q_{\mathrm{N}}^{{\mathrm{Sto}}}$$

where *Q*_N_ is total N quota (per cellular C: mol N (mol C)^−1^), the terms on the right-hand side are the contributions from protein, RNA, DNA, chlorophyll and N storage. We use empirically determined fixed elemental stoichiometry of macromolecules^21^ (Extended Data Table 1) to connect the macromolecular contributions of different elements (here C and P):

3 $$Q_{\mathrm{N}} = Q_{\mathrm{C}}^{{\mathrm{Pro}}}Y_{{\mathrm{Pro}}}^{{\mathrm{N:C}}} + Q_{\mathrm{P}}^{{\mathrm{RNA}}}Y_{{\mathrm{RNA}}}^{{\mathrm{N:P}}} + Q_{\mathrm{C}}^{{\mathrm{DNA}}}Y_{{\mathrm{DNA}}}^{{\mathrm{N:C}}} + Q_{\mathrm{C}}^{{\mathrm{Chl}}}Y_{{\mathrm{Chl}}}^{{\mathrm{N:C}}} + Q_{\mathrm{N}}^{{\mathrm{Nsto}}}$$

Here $Y_l^{j:k}$ represents the imposed elemental ratio (elements *j* and *k*) for each macromolecular pool (*l*). $Q_{\mathrm{C}}^x$ and $Q_{\mathrm{P}}^x$ describe the contributions of macromolecule *x* to the total C quota (mol C (mol C)^−1^) and P quota (mol P (mol C)^−1^), respectively.

CFM-Phyto uses the following empirically supported relationship to describe $Q_{\mathrm{P}}^{{\mathrm{RNA}}}$ (ref. ^21^):

4 $$Q_{\mathrm{P}}^{{\mathrm{RNA}}} = A_{{\mathrm{RNA}}}^{\mathrm{P}}\mu Q_{\mathrm{C}}^{{\mathrm{Pro}}} + Q_{{\mathrm{P,min}}}^{{\mathrm{RNA}}}$$

where $A_{{\mathrm{RNA}}}^{\mathrm{P}}$ is constant (below, *A* values represent constant except $A_{{\mathrm{Chl}}}$; see below), *μ* is growth rate (d^−1^) and $Q_{{\mathrm{P,min}}}^{{\mathrm{RNA}}}$ represents the minimum amount of RNA in phosphorus per cellular C (mol P (mol C)^−1^). Substituting this equation into equation (3) gives:

5 $$\begin{array}{l}Q_{\mathrm{N}} = Q_{\mathrm{C}}^{{\mathrm{Pro}}}Y_{{\mathrm{Pro}}}^{{\mathrm{N:C}}} + \left( {A_{{\mathrm{RNA}}}^{\mathrm{P}}\mu Q_{\mathrm{C}}^{{\mathrm{Pro}}} + Q_{{\mathrm{P,min}}}^{{\mathrm{RNA}}}} \right)\\Y_{{\mathrm{RNA}}}^{{\mathrm{N:P}}} + Q_{\mathrm{C}}^{{\mathrm{DNA}}}Y_{{\mathrm{DNA}}}^{{\mathrm{N:C}}} + Q_{\mathrm{C}}^{{\mathrm{Chl}}}Y_{{\mathrm{Chl}}}^{{\mathrm{N:C}}} + Q_{\mathrm{N}}^{{\mathrm{Nsto}}}\end{array}$$

In CFM-Phyto, we resolve three types of protein, photosynthetic, biosynthetic and other:

6 $$Q_{\mathrm{C}}^{{\mathrm{Pro}}} = Q_{\mathrm{C}}^{{\mathrm{Pro}}\_{\mathrm{Pho}}} + Q_{\mathrm{C}}^{{\mathrm{Pro}}\_{\mathrm{Bio}}} + Q_{\mathrm{C}}^{{\mathrm{Pro}}\_{\mathrm{Other}}}$$

Photosynthetic proteins represent those in chloroplasts largely responsible for light harvesting and electron transport. The model assumes a constant composition of chloroplasts; thus, the amount of photosynthetic protein is proportional to the amount of chlorophyll^21^:

7 $$Q_{\mathrm{C}}^{{\mathrm{Pro}}\_{\mathrm{Pho}}} = A_{{\mathrm{Pho}}}Q_{\mathrm{C}}^{{\mathrm{Chl}}}$$

Biosynthetic proteins represent proteins related to producing new material such as proteins, carbohydrates, lipids, RNAs, DNAs and other molecules. The models use the following empirically derived relationship^21^:

8 $$Q_{\mathrm{C}}^{{\mathrm{Pro}}\_{\mathrm{Bio}}} = A_{{\mathrm{Bio}}}\mu$$

Substituting equations (6)–(8) (in this order) into equation (5) leads to the following equation:

9 $$\begin{array}{l}Q_{\mathrm{N}} = \left( {A_{{\mathrm{Pho}}}Q_{\mathrm{C}}^{{\mathrm{Chl}}} + A_{{\mathrm{Bio}}}\mu + Q_{\mathrm{C}}^{{\mathrm{Pro}}\_{\mathrm{Other}}}} \right)Y_{{\mathrm{Pro}}}^{{\mathrm{N:C}}}\\ + \left( {A_{{\mathrm{RNA}}}^{\mathrm{P}}\mu \left( {A_{{\mathrm{Pho}}}Q_{\mathrm{C}}^{{\mathrm{Chl}}} + A_{{\mathrm{Bio}}}\mu + Q_{\mathrm{C}}^{{\mathrm{Pro}}\_{\mathrm{Other}}}} \right) + Q_{{\mathrm{P,min}}}^{{\mathrm{RNA}}}} \right)Y_{{\mathrm{RNA}}}^{{\mathrm{N:P}}}\\ + Q_{\mathrm{C}}^{{\mathrm{DNA}}}Y_{{\mathrm{DNA}}}^{{\mathrm{N:C}}} + Q_{\mathrm{C}}^{{\mathrm{Chl}}}Y_{{\mathrm{Chl}}}^{{\mathrm{N:C}}} + Q_{\mathrm{N}}^{{\mathrm{Sto}}}\end{array}$$

Empirically, chlorophyll depends on the growth rate and equation (10) accurately describes the relationship between the growth-rate dependences of chlorophyll under different light intensities^21^:

10 $$Q_{\mathrm{C}}^{{\mathrm{Chl}}} = A_{{\mathrm{Chl}}}\mu + B_{{\mathrm{Chl}}}$$

with $A_{{\mathrm{Chl}}} = \left( {1 + E} \right)/v_I$ and $B_{Chl} = m/v_I$ with *E* (dimensionless) as a constant representing growth-rate-dependent respiration, and *m* (d^−1^) describing maintenance respiration. *v*_*I*_ (mol C (mol C in Chl)^−1^ d^−1^) represents chlorophyll-specific photosynthesis rate based on an established function of light intensity *I* (μmol m^−2^ s^−1^)^21,57^:

11 $$v_I = v_I^{{\mathrm{max}}}\left( {1 - e^{A_II}} \right)$$

where $v_I^{{\mathrm{max}}}$ is the maximum chlorophyll-specific photosynthesis rate, *e* is the natural base and *A*_*I*_ is a combined coefficient for absorption cross-section and turnover time. Substitution of equation (10) into equation (9) leads to the following quadratic relationship between *Q*_N_ and *μ*:

12 $$Q_{\mathrm{N}} = a_{\mathrm{N}}\mu ^2 + b_{\mathrm{N}}\mu + c_{\mathrm{N}} + Q_{\mathrm{N}}^{{\mathrm{Sto}}}$$

where

$$\begin{array}{l}a_{\mathrm{N}} = A_{{\mathrm{RNA}}}^{\mathrm{P}}\left( {A_{{\mathrm{Pho}}}A_{{\mathrm{Chl}}} + A_{{\mathrm{Bio}}}} \right)Y_{{\mathrm{RNA}}}^{{\mathrm{N:P}}}\\ b_{\mathrm{N}} = \left( {A_{{\mathrm{Pho}}}A_{{\mathrm{Chl}}} + A_{{\mathrm{Bio}}}} \right)Y_{{\mathrm{Pro}}}^{{\mathrm{N:C}}} + A_{{\mathrm{Chl}}}Y_{{\mathrm{Chl}}}^{{\mathrm{N:C}}} + A_{{\mathrm{RNA}}}^{\mathrm{P}}\left( {A_{{\mathrm{Pho}}}B_{{\mathrm{Chl}}} + Q_{\mathrm{C}}^{{\mathrm{Pro}}\_{\mathrm{Other}}}} \right)Y_{\mathrm{{RNA}}}^{{\mathrm{N:P}}}\\ c_{\mathrm{N}} = B_{{\mathrm{Chl}}}Y_{{\mathrm{Chl}}}^{{\mathrm{N:C}}} + \left( {A_{{\mathrm{Pho}}}B_{{\mathrm{Chl}}} + Q_{\mathrm{C}}^{{\mathrm{Pro}}\_{\mathrm{Other}}}} \right)Y_{{\mathrm{Pro}}}^{{\mathrm{N:C}}}\\ + Q_{{\mathrm{P}},{\mathrm{min}}}^{{\mathrm{RNA}}}Y_{{\mathrm{RNA}}}^{{\mathrm{N:P}}} + Q_{\mathrm{C}}^{{\mathrm{DNA}}}Y_{{\mathrm{DNA}}}^{{\mathrm{N:C}}}\end{array}$$

### Relating P quota and growth rate

Similarly, CFM-Phyto describes the relationship between the current P quota *Q*_P_ and *μ*. P is allocated to its major molecular reservoirs:

13 $$Q_{\mathrm{P}} = Q_{\mathrm{P}}^{{\mathrm{RNA}}} + Q_{\mathrm{C}}^{{\mathrm{DNA}}}Y_{{\mathrm{DNA}}}^{{\mathrm{P:C}}} + Q_{\mathrm{P}}^{{\mathrm{Thy}}} + Q_{\mathrm{P}}^{{\mathrm{Other}}} + Q_{\mathrm{P}}^{{\mathrm{Sto}}}$$

Similar to equation (7), with the assumption of the constant composition of photosynthetic apparatus, the model connects the amount of the chlorophyll to phosphorus in thylakoid membranes:

14 $$Q_{\mathrm{P}}^{{\mathrm{Thy}}} = A_{{\mathrm{Pho}}}^{{\mathrm{P:Chl}}}Q_{\mathrm{C}}^{{\mathrm{Chl}}}$$

As for N allocation, substitution of equations (14), (4), (6), (7), (8) and (10) (in this order) into equation (13) leads to a quadratic relationship between *Q*_P_ and *μ*:

15 $$Q_{\mathrm{P}} = a_{\mathrm{P}}\mu ^2 + b_{\mathrm{P}}\mu + c_{\mathrm{P}} + Q_{\mathrm{P}}^{{\mathrm{Sto}}}$$

where

$$\begin{array}{l}a_{\mathrm{P}} = A_{{\mathrm{RNA}}}^{\mathrm{P}}\left( {A_{{\mathrm{Pho}}}A_{{\mathrm{Chl}}} + A_{{\mathrm{Bio}}}} \right)\\ b_{\mathrm{P}} = A_{{\mathrm{RNA}}}^{\mathrm{P}}\left( {A_{{\mathrm{Pho}}}B_{{\mathrm{Chl}}} + Q_{\mathrm{C}}^{{\mathrm{Pro}}\_{\mathrm{Other}}}} \right)Y_{{\mathrm{RNA}}}^{{\mathrm{N:P}}} + A_{{\mathrm{Pho}}}^{{\mathrm{P:Chl}}}A_{{\mathrm{Chl}}}\\ c_{\mathrm{P}} = Q_{{\mathrm{P,min}}}^{{\mathrm{RNA}}} + Q_{\mathrm{C}}^{{\mathrm{DNA}}}Y_{{\mathrm{DNA}}}^{{\mathrm{P:C}}} + A_{{\mathrm{Pho}}}^{{\mathrm{P:Chl}}}B_{{\mathrm{Chl}}} + Q_{\mathrm{P}}^{{\mathrm{Other}}}\end{array}$$

### Relating C quota and growth rate

Similarly, CFM-Phyto describes C allocation as follows:

16 $$\begin{array}{l}Q_{\mathrm{C}} = 1 = Q_{\mathrm{C}}^{{\mathrm{Pro}}} + Q_{\mathrm{C}}^{{\mathrm{RNA}}} + Q_{\mathrm{C}}^{{\mathrm{DNA}}} + Q_{\mathrm{C}}^{{\mathrm{Other}}} + Q_{\mathrm{C}}^{{\mathrm{Plip}} - {\mathrm{Thy}}}\\\qquad\ + Q_{\mathrm{C}}^{{\mathrm{Csto}}} + Q_{\mathrm{C}}^{{\mathrm{Nsto}}}\end{array}$$

where Plip−Thy indicates P lipid in thylakoid membranes. The equation represents the allocation per total cellular C in mol C (mol C)^−1^, so the sum of the macromolecules in C (*Q*_C_) becomes 1. Using the imposed elemental ratios of macromolecular pools ($Y_l^{j:k}$) we relate the elemental contributions:

17 $$Q_{\mathrm{C}} = Q_{\mathrm{C}}^{{\mathrm{Pro}}} + Q_{\mathrm{P}}^{{\mathrm{RNA}}}Y_{{\mathrm{RNA}}}^{{\mathrm{C:P}}} + Q_{\mathrm{C}}^{{\mathrm{DNA}}} + Q_{\mathrm{C}}^{{\mathrm{Other}}} + Q_{\mathrm{P}}^{{\mathrm{Thy}}}Y_{{\mathrm{Plip}}}^{{\mathrm{C:P}}} + Q_{\mathrm{C}}^{{\mathrm{Sto}}} + Q_{\mathrm{N}}^{{\mathrm{Sto}}}Y_{{\mathrm{Nsto}}}^{{\mathrm{C:N}}}$$

Following the steps similar to those for the N and P allocations, substituting equations (14), (4), (6), (7), (8) and (10) (in this order) into equation (17) leads to the following quadratic relationship between cellular C quota *Q*_C_ (=1 mol C (mol C)^−1^) and *μ*:

18 $$Q_{\mathrm{C}} = a_{\mathrm{C}}\mu ^2 + b_{\mathrm{C}}\mu + c_{\mathrm{C}} + Q_{\mathrm{C}}^{{\mathrm{Sto}}} + Q_{\mathrm{N}}^{{\mathrm{Sto}}}Y_{{\mathrm{Nsto}}}^{{\mathrm{C:N}}}$$

where

$$\begin{array}{l}a_{\mathrm{C}} = A_{{\mathrm{RNA}}}^{\mathrm{P}}\left( {A_{{\mathrm{Pho}}}A_{{\mathrm{Chl}}} + A_{{\mathrm{Bio}}}} \right)Y_{{\mathrm{RNA}}}^{{\mathrm{C:P}}}\\ b_{\mathrm{C}} = A_{{\mathrm{Chl}}}\left( {1 + A_{{\mathrm{Pho}}} + A_{{\mathrm{Pho}}}^{{\mathrm{P:Chl}}}Y_{{\mathrm{Plip}}}^{{\mathrm{C:P}}}} \right) + A_{{\mathrm{Bio}}} + A_{{\mathrm{RNA}}}^{\mathrm{P}}\left( {A_{{\mathrm{Pho}}}B_{{\mathrm{Chl}}} + Q_{\mathrm{C}}^{{\mathrm{Pro}}\_{\mathrm{Other}}}} \right)Y_{{\mathrm{RNA}}}^{{\mathrm{C:P}}}\\ c_{\mathrm{C}} = \left( {1 + A_{{\mathrm{Pho}}} + A_{{\mathrm{Pho}}}^{{\mathrm{P:Chl}}}Y_{{\mathrm{Plip}}}^{{\mathrm{C:P}}}} \right)B_{{\mathrm{Chl}}} + Q_{\mathrm{C}}^{{\mathrm{Pro}}\_{\rm{Other}}}\\ + Q_{{\mathrm{P}},{\mathrm{min}}}^{{\mathrm{RNA}}}Y_{{\mathrm{RNA}}}^{{\mathrm{C:P}}} + Q_{\mathrm{C}}^{{\mathrm{DNA}}} + Q_{\mathrm{C}}^{{\mathrm{Other}}}\end{array}$$

### Relating Fe quota and growth rate

In order to capture global scale biogeochemical dynamics including the iron-limited high-nitrogen, low chlorophyll regimes, CFM-Phyto^21^ is extended to resolve Fe quota and allocation. The model is guided by a laboratory proteomic study^58^ in which the major Fe allocations are to photosystems, storage and nitrogen-fixing enzymes (nitrogenase). As we do not resolve nitrogen-fixing organisms here, Fe allocation (mol Fe (mol C)^−1^) represents only the first two:

19 $$Q_{{\mathrm{Fe}}} = Q_{{\mathrm{Fe}}}^{{\mathrm{Pho}}} + Q_{{\mathrm{Fe}}}^{{\mathrm{Sto}}}$$

As for equation (7) and equation (14), we relate the allocation of Fe to photosystems to the investment in chlorophyll, $Q_{\mathrm{C}}^{{\mathrm{Chl}}}$:

20 $$Q_{{\mathrm{Fe}}}^{{\mathrm{Pho}}} = A_{{\mathrm{Pho}}}^{{\mathrm{Fe}}}Q_{\mathrm{C}}^{{\mathrm{Chl}}}$$

This is a strong simplification because the pigment to photosystem ratio is observed to vary^59^, but enables an explicit, mechanistically motivated representation of Fe limitation, which, a posteriori, results in global scale regimes of iron limitation that resemble those observed^43^ (Extended Data Fig. 4). With equations (10), (19) and (20), we obtain the following relationship between *Q*_Fe_ and *μ*:

21 $$Q_{{\mathrm{Fe}}} = A_{{\mathrm{Pho}}}^{{\mathrm{Fe}}}A_{{\mathrm{Chl}}}\mu + A_{{\mathrm{Pho}}}^{{\mathrm{Fe}}}B_{{\mathrm{Chl}}} + Q_{{\mathrm{Fe}}}^{{\mathrm{Sto}}}$$

### Evaluating the growth rate

We assume that the cellular growth rate is constrained by the most limiting element within the cell (and its associated functional macromolecules). Thus, at each time step and location, and for each cell type, the evaluation of growth rate is based on the following two steps: (1) computation of the growth rate for each element without storage; that is, the case when all of the elemental quotas are allocated to functional macromolecules; and (2) selection of the lowest growth rate among these; Liebig’s Law of the Minimum. For the first step, we define $\mu _i$ (*i* = C, N, P, Fe) as the growth rate, assuming that nutrient *i* is limiting. Under this condition, $Q_i^{{\mathrm{Sto}}}$ should be small as element *i* is allocated to other essential molecules. We assume that $Q_{\mathrm{N}}^{{\mathrm{Sto}}}$ is also small under C limitation because N storage molecules are rich in carbon. With these assumptions, the solution for $\mu _i$ is obtained by solving the standard quadratic relationships of equations (12), (15) and (18) for N, P and C, respectively, neglecting any $Q_i^{{\mathrm{Sto}}}$ terms:

22 $$\mu _i = \frac{{ - b_i + \sqrt {b_i^2 - 4a_i\left( {c_i - Q_i} \right)} }}{{2a_i}}$$

where *Q*_C_ = 1. For *μ*_Fe_, equation (21) without $Q_{{\mathrm{Fe}}}^{{\mathrm{Sto}}}$ leads to

23 $$\mu _{{\mathrm{Fe}}} = \frac{{Q_{{\mathrm{Fe}}} - A_{{\mathrm{Pho}}}^{{\mathrm{Fe}}}B_{{\mathrm{Chl}}}}}{{A_{{\mathrm{Pho}}}^{{\mathrm{Fe}}}A_{{\mathrm{Chl}}}}}$$

Once the *μ*_*i*_ values are obtained, we determine the effective growth rate, *μ*, based on the lowest value, which identifies the limiting element based on current intracellular quotas:

24 $$\mu = {\mathrm{min}}\left( {\mu _{\mathrm{N}},\mu _{\mathrm{P}},\mu _{\mathrm{C}},\mu _{{\mathrm{Fe}}}} \right)$$

### Evaluating nutrient storage

In CFM-Phyto, non-limiting nutrients can be stored in an intracellular reserve^21^, reflecting commonly observed luxury uptake. Storage is evaluated as the difference between the total elemental quota (updated later) and the functionally allocated portion of that element:

25 $$Q_i^{{\mathrm{Sto}}} = Q_i - Q_i^{{\mathrm{Non}}\_{\mathrm{Sto}}}$$

Here $Q_i^{{\mathrm{Non}}\_{\mathrm{Sto}}}$ represents the contribution to element *i* by functional, non-storage molecules. For N, P and C, $Q_i^{{\mathrm{Non}}\_{\mathrm{Sto}}}$ is represented by the non-$Q_i^{{\mathrm{Sto}}}$ terms on the right-hand side in equations (12), (15) and (18), respectively:

26 $$Q_i^{{\mathrm{Non}}\_{\mathrm{Sto}}} = a_i\mu ^2 + b_i\mu + c_i$$

Similarly, for Fe, from equation (21):

27 $$Q_{{\mathrm{Fe}}}^{{\mathrm{Non}}\_{\mathrm{Sto}}} = A_{{\mathrm{Pho}}}^{{\mathrm{Fe}}}A_{{\mathrm{Chl}}}\mu + A_{{\mathrm{Pho}}}^{{\mathrm{Fe}}}B_{{\mathrm{Chl}}}$$

When there is N storage, $Q_{\mathrm{C}}^{{\mathrm{Sto}}}$ must be recomputed to consider the allocation of C to it:

28 $$Q_{\mathrm{C}}^{{\mathrm{Sto}}} = Q_{\mathrm{C}} - Q_{\mathrm{C}}^{{\mathrm{Non}}\_{\mathrm{Sto}}} - Q_{\mathrm{N}}^{{\mathrm{Sto}}}Y_{{\mathrm{Nsto}}}^{{\mathrm{C:N}}}$$

### Evaluating the excess nutrient

Storage capacity for any element is finite and we define excess nutrient as a nutrient (N, P, Fe) that is in beyond an empirically informed, imposed maximum phytoplankton storage capacity. Excess nutrient is assumed to be excreted (see below). Excess of element *i* ($Q_i^{{\mathrm{Exc}}}$) is computed:

29 $$Q_i^{{\mathrm{Exc}}} = {\mathrm{max}}\left( {Q_i - Q_i^{{\mathrm{max}}},0} \right)$$

where $Q_i^{{\mathrm{max}}}$ is maximum capacity for nutrient *i*. For N, CFM-Phyto computes $Q_i^{{\mathrm{max}}}$ as a sum of non-storage molecules and prescribed maximum nutrient storing capacity according to model–data comparison^21^:

30 $$Q_i^{{\mathrm{max}}} = Q_i^{{\mathrm{Non}}\_{\mathrm{Sto}}} + Q_i^{{\mathrm{Sto}}\_{\mathrm{max}}}$$

Laboratory studies suggest that when P is not limiting, the phosphorus quota maximizes to a value that is almost independent of growth rate^21,39,44^. Storage of each element is finite and the upper limit to storage is imposed by specifying the maximum cellular quotas ($Q_{\mathrm{P}}^{{\mathrm{max}}}$ (ref. ^21^) and also $Q_{{\mathrm{Fe}}}^{{\mathrm{max}}}$) with size and taxonomic dependencies (for example, refs. ^27,41^). Thus, the maximum storage is represented by the difference between the prescribed maximum quota and the actual quota^21^:

31 $$Q_i^{{\mathrm{Sto}}\_{\mathrm{max}}} = Q_i^{{\mathrm{max}}} - Q_i$$

In the case where $Q_i^{{\mathrm{Sto}}}$ computed in the previous section exceeds $Q_i^{{\mathrm{Sto}}\_{\mathrm{max}}}$, the value of $Q_i^{{\mathrm{Sto}}}$ is replaced by $Q_i^{{\mathrm{Sto}}\_{\mathrm{max}}}$ and the difference is placed in the excess pool, $Q_i^{{\mathrm{Exc}}}$.

### Computing effective nutrient uptake rate

One factor that influences the cellular elemental quota is the effective nutrient uptake rate (mol *i* (mol C)^−1^ d^−1^) of N, P and Fe, which we define as follows:

32 $$V_i^{{\mathrm{Eff}}} = V_i - \frac{{Q_i^{{\mathrm{Exc}}}}}{{\tau _i^{{\mathrm{Exu}}}}}$$

where *V*_*i*_ (mol *i* (mol C)^−1^ d^−1^) is nutrient uptake rate and the second term represents the exudation of the excess nutrient based on the timescale $\tau _i^{{\mathrm{Exu}}}$ (d^−1^). For *V*_*i*_, we use Monod kinetics^60,61^:

33 $$V_i = V_i^{{\mathrm{max}}}\frac{{[i]}}{{\left[ i \right] + K_i}}$$

where $V_i^{{\mathrm{max}}}$ is maximum nutrient uptake, [*i*] (mmol m^−3^) is the environmental concentration of nutrient *i* and *K*_*i*_ (mmol m^−3^) is the half-saturation constant of *i*. Previous models have resolved the relationship between nutrient uptake and allocation to transporters^31,62^. Here we do not explicitly resolve allocation to transporters, as proteomic studies indicate that it is a relatively minor component of the proteome compared with photosystems and biosynthesis in phytoplankton^63^. Transporter proteins could be represented in a model with a finer-scale resolution of the proteome^64^.

### Differentiating small and large phytoplankton

In this model, ‘small’ phytoplankton broadly represent picocyanobacteria, which have high nutrient affinities and low maximum growth rates (for example, *Prochlorococcus*), whereas ‘large’ phytoplankton represent eukaryotes with higher maximum growth rates (for example, diatoms). The former are associated with a gleaner strategy adapted to oligotrophic regimes, while the latter are opportunistic, adapted to variable and nutrient-enriched regimes. To encapsulate this, the large phytoplankton have overall higher imposed $V_i^{{\mathrm{max}}}$ (~*µ*^max^*Q*_*i*_), *K*_*i*_ and $v_I^{\mathrm{max}}$ than for the small phytoplankton (Extended Data Table 1), consistent with the previous models (for example, ref. ^10^). In addition, the larger cells are assigned a higher $Q_{\mathrm{P}}^{{\mathrm{max}}}$ following the observed trends (Fig. 1 and Extended Data Table 1).

### Computing the change in the elemental stoichiometry

The computation of the change in the elemental quotas is done based on the balance between the effective nutrient uptake rate and the loss of nutrient to the new cells:

34 $$\frac{{{\mathrm{d}}Q_i}}{{{\mathrm{d}}t}} = V_i^{{\mathrm{Eff}}} - \mu Q_i$$

This change in the elemental quotas based on the cellular processes and the passive transport of elements in phytoplankton by the flow field created by MITgcm governs the elemental stoichiometry of the next time step at a specific grid box, as in other versions of ecological models with MITgcm^10^.

### Calculation of CV values

We computed the CV values based on the following equation:

35 $${\mathrm{CV}} = \frac{\sigma }{{\bar x}}$$

where *σ* is the standard deviation and $\bar x$ is the mean. The purpose of this computation is to quantify the latitudinal variation of the averaged elemental stoichiometry. Thus, we used the averaged values for each latitude (as plotted in Fig. 2) for the calculation of *σ* and $\bar x$.

### MITgcm-CFM

The biogeochemical and ecological component of the model resolves the cycling of C, P, N and Fe through inorganic, living, dissolved and particulate organic phases. The biogeochemical and biological tracers are transported and mixed by the MIT general circulation model (MITgcm)^35,36^, constrained to be consistent with altimetric and hydrographic observations (the ECCO-GODAE state estimates)^65^. This three-dimensional configuration has a coarse resolution (1° × 1° horizontally) and 23 depth levels ranging from 5 m at the surface to 5450 m at depth. The model was run for three years, and the results of the third year were analysed, by which time the modelled plankton distribution becomes quasi-stable. Equations for the biogeochemical processes are as described by equations and parameters in previous studies^10,38^. Here, however, we include only nitrate for inorganic nitrogen, and do not resolve the silica cycle. We simulated eukaryotic and prokaryotic analogues of phytoplankton (as ‘large’ and ‘small’ phytoplankton). The eukaryotic analogue has a higher maximum C fixation rate for the same macromolecular composition and higher maximum nutrient uptake rates, but also has overall higher half-saturation constants for nutrient uptake. We used light absorption spectra of picoeukaryotes, which sits in-between small prokaryotes and large eukaryotes^10^. In MITgcm, the mortality of phytoplankton is represented by the sum of a linear term (*m*_l_), representing sinking and maintenance losses, and quadratic terms representing the action of unresolved next-trophic levels^66,67^, implicitly assuming that the higher-trophic-level biomass scales with that of its prey. We assumed that the latter term is small to avoid introducing additional uncertainties. Similarly, we do not resolve the stoichiometric effects of prey selection due to the nutritional status of prey, or viral partitioning of nutrients in the environment^50^. Atmospheric iron deposition varies by orders of magnitude around the globe and has a large margin of uncertainty, including the bio-availability of the deposited iron, which in turn depends on the source and chemical history of the deposited material^68^. To realize a realistic global net primary production, we doubled the atmospheric iron input from ref. ^10^; this factor is well within the uncertainty of the iron supply estimates. Each of the two phytoplankton groups has variable C:N:P:Fe as determined by the component macromolecules at each time step. The pools of C, N, P and Fe are tracked within the modelled three-dimensional flow fields.

## Online content

Any methods, additional references, Nature Research reporting summaries, source data, extended data, supplementary information, acknowledgements, peer review information; details of author contributions and competing interests; and statements of data and code availability are available at 10.1038/s41561-022-01066-2.

### Supplementary information

Supplementary Data 1 Stoichiometric data compilation from multiple studies.

## Figures and Tables

**Extended Data Fig. 5:**
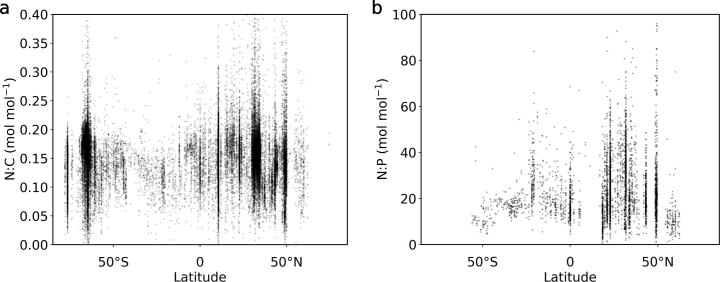
Distribution of the elemental ratios measured in particulate organic matter from global field data. (A) N:C. (B) N:P. A small fraction of data (<1%) lies outside of the plotted range. Data are based on the global compilation^52,53^.

**Extended Data Table 1:**
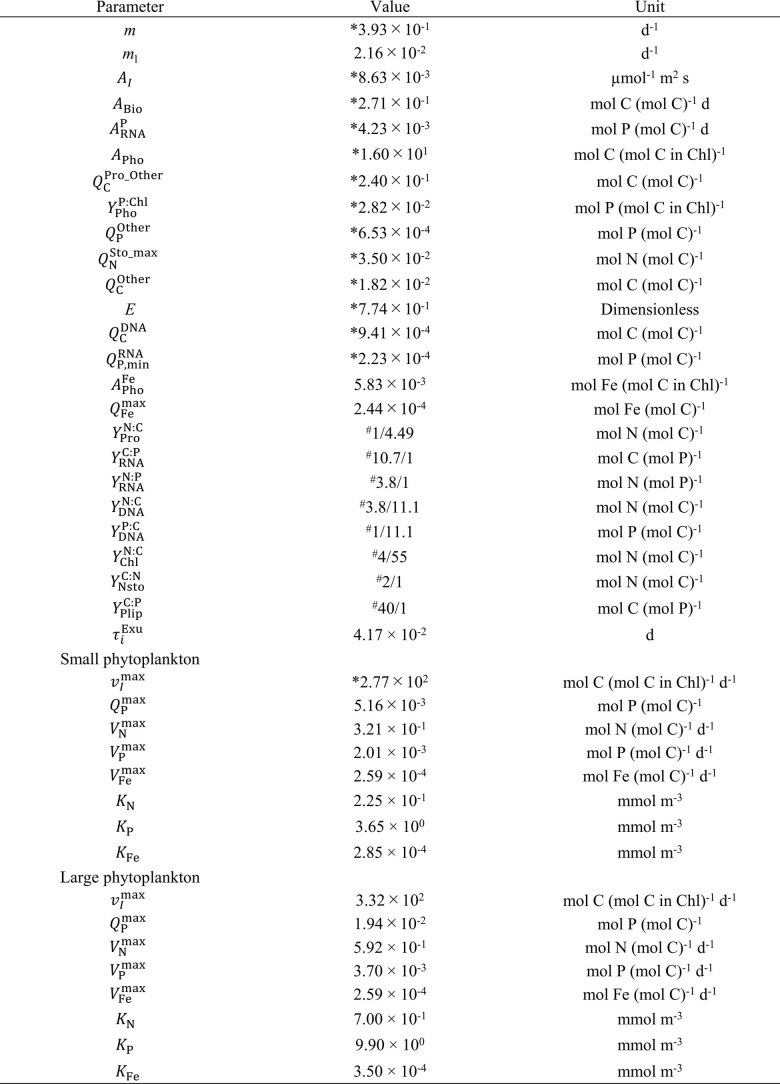
Parameter values

-: Dimensionless (for example, mol mol^−1^), *Values from the original CFM-Phyto study^21^, ^#^Empirical stoichiometric parameters^21^.

The rest of the parameters were chosen to reproduce realistic nutrient limitation, reasonable net primary production, and observed magnitude of elemental stoichiometry. We have given overall higher *V*^*max*^ and *K* values for large phytoplankton as typically modeled (for example, ref. ^10^), and higher $Q_P^{max}$ for large phytoplankton based on observations^26^.

## Data Availability

The model output from this study is available at 10.6084/m9.figshare.21197578.

## Code availability

The physical model and ecosystem code are available at http://mitgcm.org and https://gitlab.com/darwinproject/gud, respectively, and the specific modification for this study can be downloaded from https://zenodo.org/record/4730684.
